# A person-reported cumulative social risk measure does not show bias by income and education

**DOI:** 10.1186/s41687-024-00772-2

**Published:** 2024-08-12

**Authors:** Salene M.W. Jones, Katherine J. Briant, David R. Doody, Ronaldo Iachan, Jason A. Mendoza

**Affiliations:** 1 Fred Hutchinson Cancer Center, 1100 Fairview Ave N, Mailstop M4-B402, Seattle, WA 98109 USA; 2 ICF International, Washington DC, United States

**Keywords:** Social need, Financial hardship, Financial burden, Economic wellbeing

## Abstract

**Background:**

Social risk such as housing instability, trouble affording medical care and food insecurity are a downstream effect of social determinants of health (SDOHs) and are frequently associated with worse health. SDOHs include experiences of racism, sexism and other discrimination as well as differences in income and education. The collective effects of each social risk a person reports are called cumulative social risk. Cumulative social risk has traditionally been measured through counts or sum scores that treat each social risk as equivalent. We have proposed to use item response theory (IRT) as an alternative measure of person-reported cumulative social risk as IRT accounts for the severity in each risk and allows for more efficient screening with computerized adaptive testing.

**Methods:**

We conducted a differential item functioning (DIF) analysis comparing IRT-based person-reported cumulative social risk scores by income and education in a population-based sample (*n* = 2122). Six social risk items were analyzed using the two-parameter logistic model and graded response model.

**Results:**

Analyses showed no DIF on an IRT-based cumulative social risk score by education level for the six items examined. Statistically significant DIF was found on three items by income level but the ultimate effect on the scores was negligible.

**Conclusions:**

Results suggest an IRT-based cumulative social risk score is not biased by education and income level and can be used for comparisons between groups. An IRT-based cumulative social risk score will be useful for combining datasets to examine policy factors affecting social risk and for more efficient screening of patients for social risk using computerized adaptive testing.

**Supplementary Information:**

The online version contains supplementary material available at 10.1186/s41687-024-00772-2.

## Introduction

The past decade has seen an increase in research on social determinants of health (SDoH; [1]). SDOHs are societal and community level factors that influence people’s health and include racism, sexism, housing policy and economic policy [2]. SDoHs lead to individual level effects, often called social risks or social needs [3]. These social risks can then lead to poor health [2–7]. For example, food insecurity has been associated with lower odds of cancer screening and control of diabetes [8, 9].

Individual social risks are one person-level measure of the effects of SDoHs and recent research has shown the importance of cumulative social risk [10]. For the purposes of this study, we define cumulative social risk as the combined impact of each individual social risk a person is experiencing. Cumulative social risk has been associated with earlier mortality from cancer [11]. Individual social risks are important indicators of specific needs while cumulative social risk helps assess the total stress a person is experiencing and how that stress can shift from one need to another over time.

One challenge with measuring person-reported cumulative social risk is how to combine the individual risks. Social risks are usually assessed using self-report surveys or questionnaires [12–15]. Questionnaires can be scored either as sum scores with a numerical value assigned to each response option or as count scores with each reported social risk counted as present or absent [10, 11, 16]. One problem with using sum or count scores for cumulative social risk is that each risk is treated equally when clearly of distinct severities. For example, worry about not being able to pay rent would be treated as equivalent to not having working smoke detectors in sum and count scores. Instead, we have proposed the use of item response theory (IRT) to create cumulative social risk scores and weight each risk by severity [17, 18].

Another benefit of IRT is the ability to detect potential bias in cumulative social risk scores, called differential item functioning (DIF) in IRT [19, 20]. Bias means a particular item on a questionnaire or survey does not reflect the construct of interest, social risk in this case, but instead reflects group membership or a different approach to answering the question between groups. For example, depression questionnaires do not include questions on crying because this symptom tends to reflect gender prescriptions on whether crying is appropriate or not. An item asking about crying would not assess different levels of severity between genders and could bias assessment. For cumulative social risk, measures should not be biased by socioeconomic status as these financial SDoH measures likely influence social risk and comparisons between socioeconomic status and cumulative social risk are needed to assess the mechanisms of policy changes and interventions. Any measure of cumulative social risk, including an IRT-based one, should not be biased by socioeconomic characteristics such as education and income.

To ensure item response theory can be used to assess cumulative social risk, this study tested for statistically significant and meaningful DIF between income and education groups on a measure of cumulative social risk. We have previously shown that using IRT to assess cumulative social risk is feasible [18] and in this study used IRT to assess for DIF when measuring cumulative social risk. A secondary analysis was conducted of a population-based, cross-sectional survey that included six questions on individual social risks. Income and education were chosen as these are some of the most commonly used measures of socioeconomic status [21]. Results could support the use of item response theory to assess cumulative social risk and lead to expanded harmonization of datasets to study the policy and community level determinants of cumulative social risk and to improve clinical screening for social risk.

## Methods

### Participants and procedures

Participants were recruited from the general population of Washington State using purchased mailing lists. Potential participants (*n* = 66,500) were sent a postcard in the mail that briefly explained the study and provided information on how to access the study online through the Research Electronic Data Capture (REDCap) platform. People who were interested went to the REDCap web address for the survey and read an informed consent statement. If the person consented to the study, they continued through to the survey. If the person did not consent and did not want to participate, they were able to close the browser window. Participants then completed the survey and received a $10 incentive. Recruitment occurred between April and December 2021. The survey was offered in both English and Spanish. Later waves of recruitment were targeted to increase representation of people from racial and ethnic minority groups and from less populous counties. Study procedures were approved by the Fred Hutchinson Cancer Center institutional review board before recruitment started. The datasets generated from this study are not publicly available due to the sensitive nature of the data.

### Measures

Six items from the survey were used in the reported study. All items had been used in prior studies. First, a general item on financial security asked participants how they were doing on their present income: living comfortably, getting by, finding it difficult, and finding it very difficult [22]. Two questions asked about housing. The first assessed whether the person had been living in stable housing for the past two months [23]. The second question asked if participants were worried about not having stable housing in the coming two months [23]. Both housing questions had yes/no response options. A single question asked if participants needed to see a doctor but could not because of cost in the past 12 months with a yes/no response option [24]. Two questions assessed food insecurity [25]. The first food insecurity question asked if participants were worried about running out of food before having money to buy more. The second question asked whether food did not last and the participant did not have money to buy more. Both food insecurity questions used a three-point response scale: never true; sometimes true; and often true. All questions were coded so higher values indicated more need.

### Statistical analyses

Item response theory (IRT) models were used to test the social risk items for bias by education and income level. IRT models differ from traditional sum scores that treat each item or social need as equivalent. IRT models estimate two parameters, slope and severity parameters, to account for the unique need reflected by each item [26]. Slope assesses how accurately the item assesses cumulative risk with higher slopes indicating more accuracy. Severity parameters indicate the level of risk (need) reflected by each item. For example, the housing worry item might reflect a moderate level of risk whereas the unstable housing item reflects a higher level of risk. IRT accounts for this difference whereas traditional sum scores would treat these as equivalent. For this study, we used the two parameter logistic model [27] for the dichotomous items and the graded response model [28] for items with three or more responses. People with missing data on one or more of the six items were included in the analysis as long as one social risk item was answered, and model parameters were estimated from the available data. Model fit was assessed with the root mean square error of approximation (RMSEA) using the guideline of RMSEA’s less than 0.08 to indicate good fit [29].

We used the IRT models to test for bias, also called differential item functioning (DIF), by education and income. Participants were divided into two groups for education: bachelor’s degree or higher and less than a bachelor’s degree. Participants were also classified into two groups by income: greater than $75,000 per year and less than $75,000 per year. We then estimated separate IRT models for each group and tested for statistically significant differences in the parameters between the groups [30]. Due to the high number of comparisons, the Benjamini Hochberg Type I error correction was used [31]. If statistically significant DIF was found, we then computed scores using IRT models accounting for the DIF and IRT models not accounting for the DIF. Intraclass correlations [32] were run between the two cumulative social risk scores to assess impact of DIF on the final scores. SPSS 28 was used for data management and cleaning and IRTPRO 5.1 was used for DIF analyses.

## Results

A total of 2137 people responded to the survey (response rate 3.2%). As shown in the supplemental material, all counties within the state were represented except one of 39 total counties and response rates did not meaningfully differ between urban and rural counties. When excluding the one county with no responses, response rates in rural counties ranged from 1.0 to 4.4%. Response rates in urban counties ranged from 2.7 to 4.8%. Fifteen people had to be excluded from these analyses because they had not answered any of the social risk questions for a final sample of 2122. Descriptive statistics for the sample are reported in Table 1. All races and ethnicities as well as all levels of income and education were represented in the survey sample. A diversity of experiences with social risk were reported, although many participants did not report any social risk.

**Table 1:** Sample characteristics (*n* = 2122)

Characteristic	*N* (%) or Mean (SD)
Age	51.62 (17.34)
Gender	
Male	1121 (52.8)
Female	978 (46.1)
Did not answer	23 (1.1)
Transgender	13 (0.6)
Race/ethnicity	
White	1779 (83.8)
Black or African American	80 (3.8)
Hispanic	272 (12.8)
American Indian or Alaska Native	72 (3.4)
Asian	136 (6.4)
Pacific Islander	18 (0.8)
Two or more races	98 (4.6)
Income	
$0 to $9,999	41 (1.9)
$10,000 to under $14,999	48 (2.3)
$15,000 to under $19,999	56 (2.6)
$20,000 to under $34,999	163 (7.7)
$35,000 to under $49,999	231 (10.9)
$50,000 to under $74,999	359 (16.9)
$75,000 to under $99,999	328 (15.5)
$100,000 to under $199,999	537 (25.3)
$200,000 or more	209 (9.8)
Missing	150 (7.1)
Education	
Less than high school graduation	34 (1.6)
High school graduate	174 (8.2)
Post high school vocational or technical training	135 (6.4)
Some college	418 (19.7)
Bachelor’s degree	730 (34.4)
Graduate degree (master’s or doctorate)	598 (28.2)
Marital status	
Married	1186 (55.9)
Living as married	100 (4.7)
Divorced	265 (12.5)
Widowed	96 (4.5)
Separated	19 (0.9)
Single, never been married	405 (19.1)
Missing	51 (2.4)
Have health insurance	
Yes	1995 (94.0)
No	85 (4.0)
Missing	42 (2.0)
Financial security	
Living comfortable on present income	1168 (55.0)
Getting by on present income	642 (30.3)
Finding it difficult on present income	183 (8.6)
Finding it very difficult on present income	74 (3.5)
Missing	55 (2.6)
Housing instability	32 (1.5)
Housing worry	116 (5.5)
Trouble with medical costs	209 (9.8)
Food insecurity, worry	
Often true	41 (1.9)
Sometimes true	196 (9.2)
Never true	1843 (86.9)
Missing	42 (2.0)
Food insecurity, last	
Often true	32 (1.5)
Sometimes true	155 (7.3)
Never true	1896 (89.3)
Missing	39 (1.8)

### Differential item functioning by education

Overall, results showed no DIF in assessing cumulative social risk between those who had a bachelor’s degree or higher and those who did not. The model for education converged in 90 iterations and had an RMSEA less than 0.01. As shown in Table 2, none of the comparisons of IRT parameters were statistically different between education groups both before the Type I error correction and after the Benjamini-Hochberg correction.

**Table 2:** Differential item function χ2 and *p*-values. Bold indicates significant after Benjamini-Hochberg type I error correction. The alpha level of 0.05 is distributed across each of the 18 tests to control for Type I error without compromising power. The alpha levels ranged from 0.001 to 0.025

Item	Education total χ^2^	Total, *p*-value	Education slope χ2	Slope, *p*-value	Education, severity χ2	Severity, *p*-value
Financial security	3.900	0.421	2.700	0.099	1.200	0.761
Housing stability	0.500	0.785	0.100	0.796	0.400	0.519
Housing worry	0.100	0.963	0.000	0.837	0.000	0.857
Medical costs	1.300	0.518	0.000	1.000	1.300	0.251
Food insecurity, worry	1.700	0.644	1.400	0.230	0.200	0.893
Food insecurity, last	0.200	0.978	0.000	0.850	0.200	0.923

Item	Income total χ2	Total, *p*-value	Income slope χ2	Slope, *p*-value	Income, severity χ2	Severity, *p*-value
Financial security	**13.900**	**0.008**	0.400	0.523	**13.500**	**0.004**
Housing stability	3.100	0.217	2.800	0.096	0.300	0.598
Housing worry	1.100	0.582	0.800	0.361	0.200	0.618
Medical costs	1.600	0.440	0.900	0.353	0.800	0.379
Food insecurity, worry	**11.800**	**0.008**	**8.100**	**0.005**	3.700	0.157
Food insecurity, last	**129.500**	**0.000**	**75.700**	**0.000**	**53.800**	**0.000**

### Differential item functioning by income

**Fig. 1:**
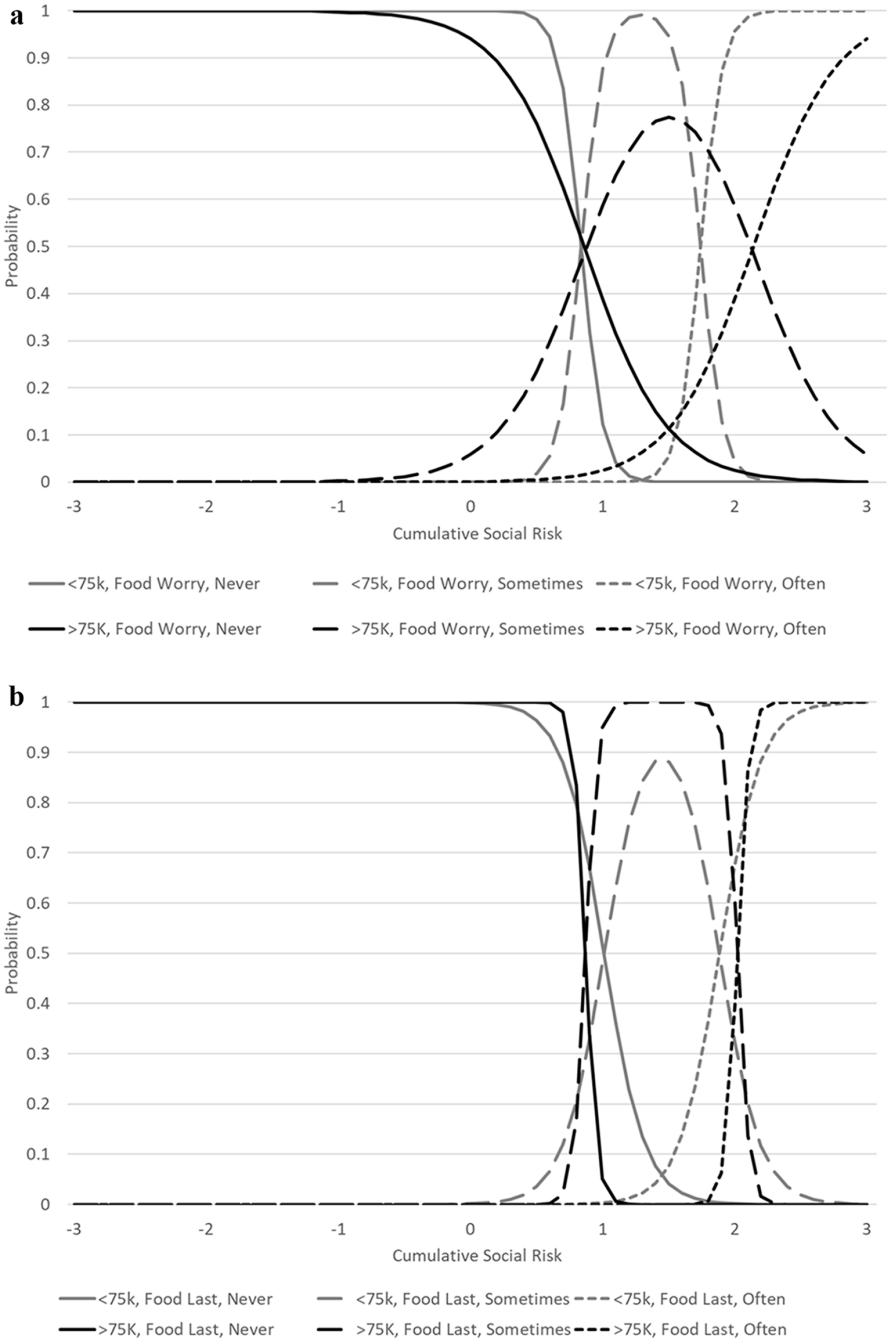
Item characteristic curves (tracelines) for food insecurity items by income. Food worry refers to the item on worry about having enough food and food last refers to food not lasting before they had money to buy more. Part a displays results for worry about affording food, part b displays results for not having food last until they could buy more

**Fig. 2:**
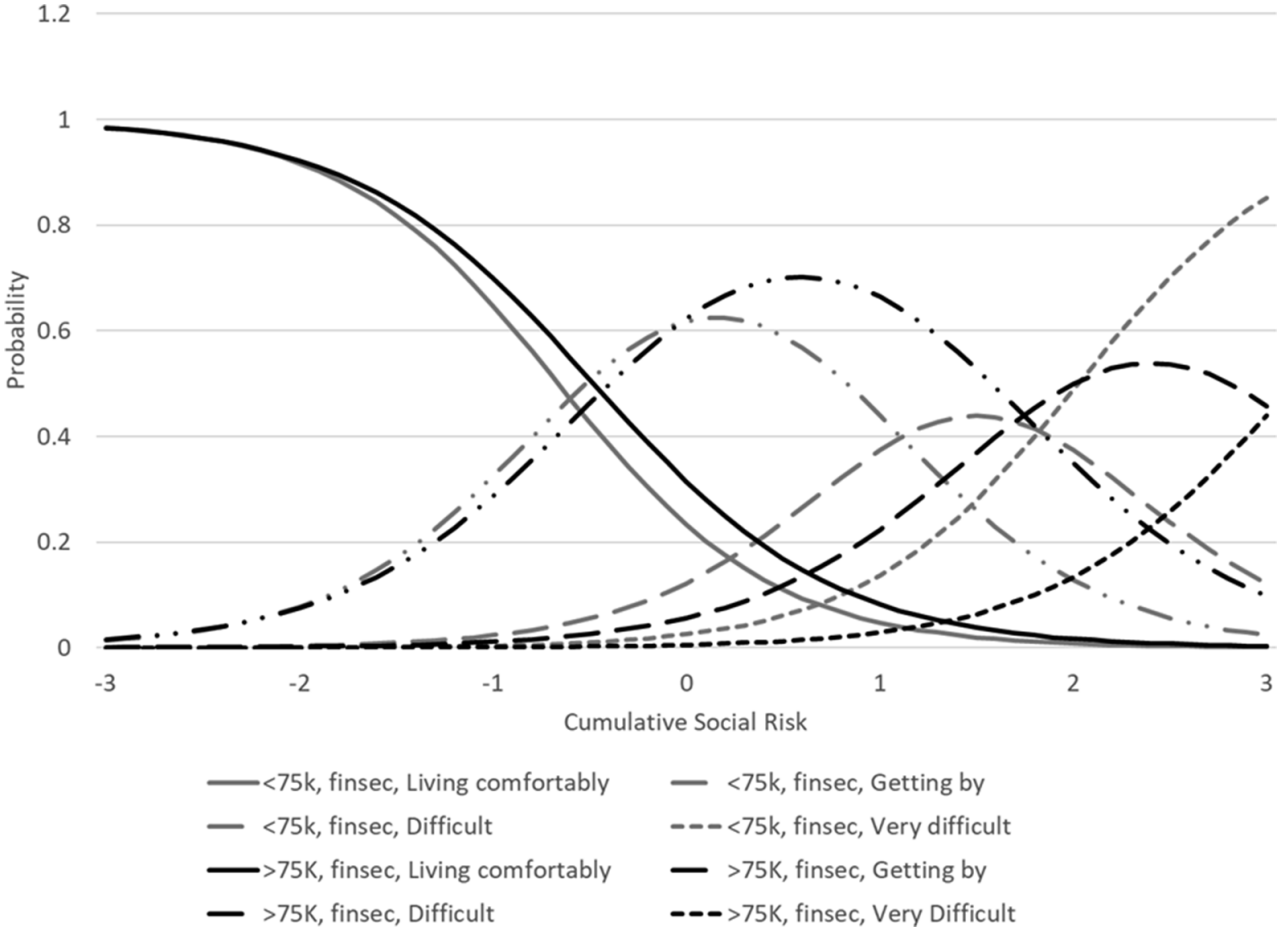
Item characteristic curves (tracelines) for general financial security items by income. 75 K=$75,000 in yearly household income

The analyses comparing income groups suggested no substantial DIF in assessing cumulative social risk. The model for income converged in 124 iterations and the RMSEA was less than 0.01. As shown in Table 2, three items had statistically significant differences, the item on financial security and the two food insecurity items. The financial security item showed differences between income groups by severity of cumulative social risk while the worry about food insecurity item showed differences by accuracy/slope. The item on food lasting showed differences by both accuracy/slope and by severity. Figure 1 shows the differences in probability of choosing each response option on the food insecurity items between the income groups and Fig. 2 shows the differences by general financial security. When comparing IRT scores accounting for DIF and not accounting for DIF, the ICC was 0.97 (95% confidence interval: 0.96, 0.97).

## Discussion

This study tested for bias between income and education groups in responding to survey items assessing cumulative social risk. Results showed no statistically significant bias between people who had a bachelor’s degree and those who do not have a bachelor’s degree. Comparisons by income found statistically significant bias on two food insecurity items and a financial security item between those with household incomes over $75,000 and those with household incomes below $75,000. However, comparisons of scores correcting for and not correcting for the bias found the difference by income to be negligible. The DIF analysis suggests that IRT can be used to calculate cumulative social risk and these IRT scores can be compared across socioeconomic groups.

The results from this study support the use of IRT for measuring cumulative social risk. Given the lack of meaningful bias by education and income, an IRT-based cumulative social risk measure could be used to assess effects of policy and individual-level SDoH interventions. Another advantage of IRT scores over sum and count scores is that respondents do not have to answer the same items [17]. IRT scores for cumulative social risk could be used to combine large national survey datasets and harmonize cumulative social risk scores on a common metric. Often these datasets may not have enough participants in each state or county to evaluate different state laws or social programs, but IRT scores could allow multiple datasets to be combined and therefore allow comparisons across smaller geographic areas with different policies.

Another advantage of IRT scores for cumulative social risk is the ability to track burden over time. Many large national surveys understandably change the questions over time to reflect the most pressing needs. For example, the National Health Interview Survey [33] used to include items on financial worries but stopped using most of those questions in 2019. This creates a problem for sum and count scores that require the exact same questions to be asked each time for longitudinal comparisons. Once an IRT scoring algorithm is created, it can be used to create comparable scores across each survey wave as long as one social risk item is included but all the other items can be different. Rather than needing to collect new data, previously collected national surveys could be harmonized using IRT cumulative social risk scores and previous policies and community factors could be evaluated. Creating an IRT scoring algorithm for cumulative social risk could allow both changing questions to fit current needs and comparisons to historical data.

The use of IRT for assessing cumulative social risk could also help healthcare systems screen for social risk more efficiently. In the United States, accreditation agencies are requiring clinics to screen patients for social risk but there is no guidance on which measures to use and many measures are long (20 + items), [15, 21, 34–37]. An IRT-based measure of cumulative social risk could be used with computerized adaptive testing to limit the number of questions each person needs to answer to just those relevant to each patient. The use of IRT for social risk screening could reduce patient and clinician burden and improve healthcare services.

The contributions of this study should be understood within the limitations. We were only able to examine DIF for two socioeconomic measures, income and education, because this was a secondary data analysis. Although the sample had good representation across racial and ethnic groups, the subgroups were not large enough to examine DIF between them. The survey was only distributed to one state and results might not generalize nationally or internationally. The survey only included six social risk items but these items spanned multiple dimensions including housing, food insecurity, medical costs and general financial wellbeing. While these limitations temper the generalizability of results, the use of a population-based sample and survey items across social risk dimensions still support the conclusion that IRT can be used to assess cumulative social risk.

The study limitations suggest directions for future research. Studies with a wider diversity of social risk items and covering more geographic areas are needed to replicate the results. Larger studies with greater representation of social risk items could also create the IRT scoring algorithm that could be used to harmonize currently available survey datasets. Future studies should also examine DIF on cumulative social risk measures by race and ethnicity as well as other socioeconomic indicators including Federal Poverty Level. These future studies could help accelerate the identification of policies and interventions to reduce cumulative social risk and address SDoHs.

## Conclusions

Assessing cumulative social risk using IRT is feasible and scores appear comparable across socioeconomic levels. IRT scores may be preferable to count and sum scores for cumulative social risk due to weighting individual risks by severity and ability to combine datasets that measured different social risks. Additional research is needed to create an IRT scoring algorithm that could help combine these datasets and allow more efficient and flexible tracking over time of cumulative social risk. Overall, IRT holds promise for accelerating research on alleviating cumulative social risk.

### Electronic supplementary material

Below is the link to the electronic supplementary material.

Supplementary Material 1

## Data Availability

The datasets generated from this study are not publicly available due to the sensitive nature of the data.
