# Supplementary material for: A person-reported cumulative social risk measure does not show bias by income and education
Source: J Patient Rep Outcomes. 2024 Aug 12;8:90. doi: 10.1186/s41687-024-00772-2 (PMC11319681; doi:10.1186/s41687-024-00772-2)

Supplemental Materials

Figure S1: Response rate by county. Underlined counties are urban. Large cities are located in the urban counties, such as Seattle in King County and Tacoma in Pierce County.


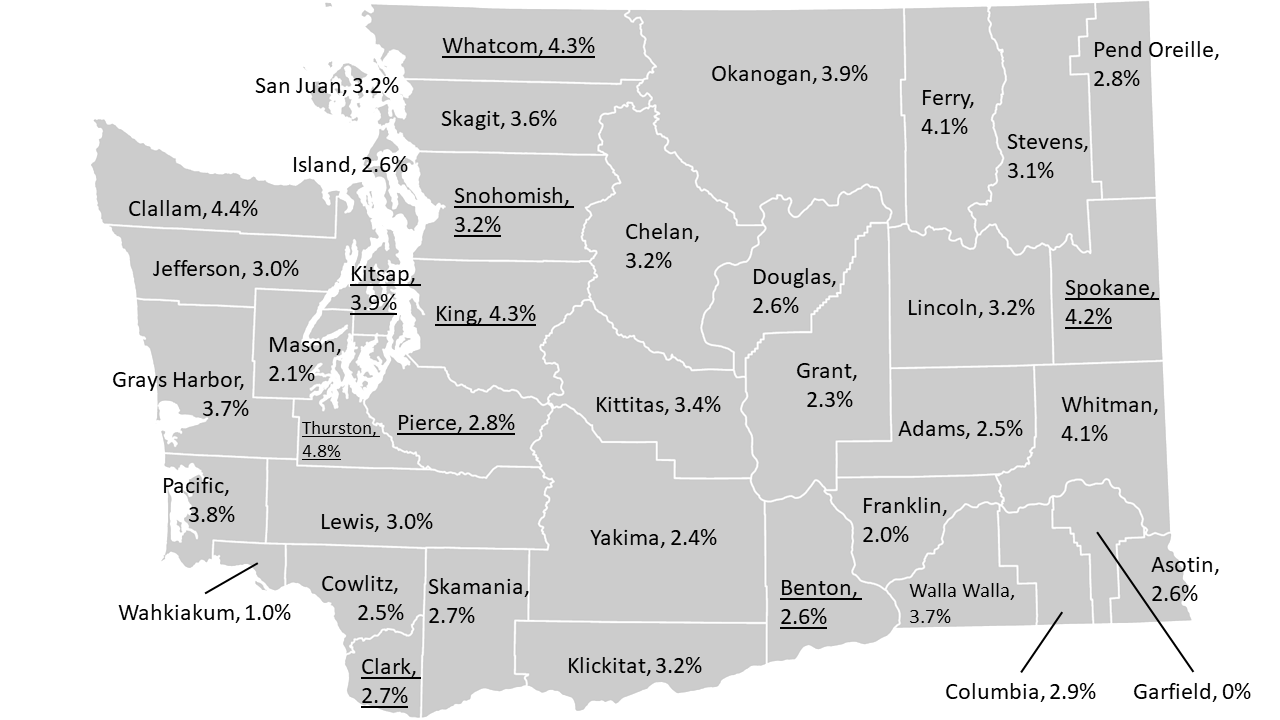

Supplement: Supplementary file 1 — Supplementary Material 1 [file 41687_2024_772_MOESM1_ESM.docx]
